# Determinants of hypertension medication adherence among older adults in a resource-limited peri-urban setting, Ghana

**DOI:** 10.3389/fpubh.2026.1848188

**Published:** 2026-07-29

**Authors:** Kennedy Diema Konlan, Charlotte Amanor, Mary Amponsah, Emma Amegashie, Lucy Amissah, Peace Dzifa Alordey, Shirley Alatiah

**Affiliations:** Department of Public Health Nursing, School of Nursing and Midwifery, University of Health and Allied Sciences, Ho, Ghana

**Keywords:** adherence, determinant, hypertension, medication, older adults

## Abstract

**Background:**

Hypertension is a leading cause of morbidity and mortality among older adults, with effective management heavily reliant on consistent medication adherence.

**Aim:**

This study aimed to evaluate the knowledge, prevalence, and associated factors influencing medication adherence among older adult hypertensive patients in Sokode, Ho.

**Methods:**

A community-based cross-sectional study was conducted among 202 older adults (≥60 years) with hypertension in Sokode. A structured questionnaire was used to collect data on sociodemographic, medical history, knowledge of hypertension and medication, adherence behaviors, and barriers to adherence. Knowledge was assessed using a Likert scale and categorized as poor, satisfactory, or excellent. Medication adherence was measured using six behavioral items and classified as good or poor. Data were analyzed using R version 4.5.1, with descriptive statistics and chi-square tests applied.

**Results:**

The majority of participants (80.2%) had good knowledge of hypertension and its management. However, medication adherence was poor: 60.9% demonstrated poor adherence. Common reasons for non-adherence included forgetfulness (46%), stopping medication when feeling better (46.5%), and cultural/religious beliefs (50.5%). Factors significantly associated with higher adherence included monthly income, age (above 79 years), and years since diagnosis (more than 10 years). Patients earning between 1000.00 and 2000.00 (aOR = 0.57, 95%CI: 0.41–0.81, *p*-value = 0.002) and those earning above 2000.00 (aOR = 0.87, 95%CI: 0.41–0.90, *p*-value = 0.002) had lower odds of adherence compared to those earning less than 1000.00. Also, older adults aged above 79 years (aOR = 2.3, 95%CI: 0.38–0.98, *p*-value = 0.050) and those diagnosed more than 10 years (aOR = 3.11, 95%CI: 1.29–1.62, *p*-value = 0.045) had higher odds of medication adherence.

**Conclusion:**

Despite adequate knowledge about hypertension, medication adherence among older adult patients in Sokode remains critically low. Interventions should move beyond general education to include practical support such as medication reminders, simplified regimens, culturally sensitive counseling, and continuous follow-up to improve adherence and health outcomes in this population.

## Introduction

Hypertension is one of the leading causes of morbidity and mortality, and is prevalent among older adults, necessitating sustained, consistent medication adherence (1, 2). Medication adherence is necessary to manage blood pressure effectively and prevent severe complications such as strokes, heart attacks, and kidney failure (3, 4). Non-adherence to hypertensive medication significantly impacts quality of life by exacerbating blood pressure control, leading to severe cardiovascular diseases and increased mortality (4, 5). Low adherence rates can result in poor health outcomes, higher healthcare costs, and diminished quality of life for older adults (6, 7). Adherence among older hypertensive patients remains critically low (8). Globally, hypertension medication non-adherence rates range from 27 to 55%, varying by region and socioeconomic status (9, 10). Medication adherence levels may be influenced by access to healthcare services and quality of interactions, ease of access, and positive relationships with health workers (11). Demographic factors like age, gender, and urban versus rural residency were associated with medication adherence; for instance, rural residents and younger older adults often display lower adherence rates, possibly due to logistical barriers and reduced health education outreach (12). Particularly in low-income countries, non-adherence prevalence is higher due to systemic barriers such as limited healthcare infrastructure and medication costs (9, 13).

The ability to manage hypertension is often influenced by various factors, including knowledge of the condition, access to medications, and family support (14, 15). Non-adherence is linked to uncontrolled hypertension, heightening risks for stroke, heart attacks, kidney failure, and all-cause mortality (16, 17). Older adults are particularly vulnerable to hypertension and its complications, including stroke, kidney disease, and heart failure (18). Among older adults, factors influencing non-adherence are multifaceted, including cognitive decline, polypharmacy, financial limitations, side effects, and poor health literacy (19). For instance, inadequate health literacy and negative illness perceptions explained up to 21.5% of non-adherence variance among older adults (17, 19). Adherence rates are often suboptimal due to factors such as forgetfulness, complex medication regimens, financial challenges, side effects, and limited health literacy. Sociodemographic factors such as retirement status, age, and history of stroke also significantly impact adherence (11, 20). Non-adherence to medication can stem from multiple factors, including forgetfulness due to age-related cognitive decline, lack of understanding of the importance of consistent medication use, financial barriers to accessing medications, and the burden of polypharmacy (21). Addressing these challenges requires a holistic approach involving education, targeted health interventions, and fostering collaboration between patients and healthcare providers to build sustainable adherence practices.

Given the current gaps in knowledge and the increasing burden of hypertension among older adults in peri-urban Ghana. Given the high prevalence of hypertension in Ghana among older adults (22), few studies have focused on medication adherence among older adults (23, 24). Yet these studies did not particularly consider peri-urban communities like Sokode. In the Sokode community, medication adherence among older adult hypertensive patients is not known, with many patients presenting with uncontrolled blood pressure despite being on prescribed medication regimens. Additionally, sociocultural factors such as reliance on traditional medicine and misconceptions about hypertension and its treatment further complicate adherence. Results of this study can provide insights into culturally sensitive and sustainable strategies to improve adherence and reduce the long-term impact of hypertension on public health systems. By focusing on hypertensives and older adults in Sokode, this study aims to identify the specific factors influencing medication adherence, laying the groundwork for tailored interventions to enhance adherence rates, improve health outcomes, and inform policy and healthcare delivery frameworks for aging populations in Ghana.

## Methods

### Study design and setting

This study adopted a cross-sectional design to examine the factors influencing medication adherence among older adults with hypertension in the Sokode community. Data were collected at a point in time, and no follow-up was required.

The study was carried out in the Sokode community of the Volta Region. Sokode is in the Ho municipality of the Volta Region of Ghana. Sokode is made up of five communities, which include Sokode-Ando, Sokode-Etoe, Sokode-Bagble, Sokode-Gborgame, and Sokode-Lokoe. The community has an estimated population of more than 40,000 people. Ho City lies between Mount Adaklu and Mount Galenukui. The Municipality shares a boundary with Adaklu and Agotime - Ziope districts to the south, Ho West District to the north and west, and the Republic of Togo to the East. Its total land area is 2,361 km^2^, thus representing 1.5% of the region’s total land area. Of the employed population, about 21.4% are engaged as skilled agricultural, forestry, and fishery workers, 26.8% are engaged in service and sales, while 22.6% are in craft and related trade, and 15.8% are engaged as managers, professionals, and technicians.

### Population and sample

The study population for this study comprised older adults aged 60 years and above who resided in Sokode. This population segment was selected due to the increased prevalence of hypertension and related health conditions among older adults, as well as their unique health-seeking behaviors influenced by socio-cultural, economic, and health system factors. Individuals aged 60 years or older who have a medical diagnosis of hypertension were selected. Adult hypertensive patients aged 60 years or older who were currently taking antihypertensive medication (or were prescribed) for at least 6 months were included. Individuals with severe cognitive impairments or other conditions that may hinder their ability to provide accurate information without a caregiver were excluded. This criterion ensured the inclusion of participants who had sufficient experience with hypertension management and medication adherence.

Using the Cochrane formulae, where 𝑛 = sample size, 𝑝 = the population, 86.8% [Woodham et al. (2018) 𝑒 = allowable sampling error (𝑒 = 0.05), confidence interval of 95% and 𝑧 = 𝑧 value at confidence interval = 1.96].


$$n=\frac{p(1-p)Z^{2}}{e^{2}},n=177$$

With a non-response rate of 10% expected, 195 hypertensive patients were supposed to be recruited for this study, but 202 patients were finally recruited.

The multistage sampling approach was employed. The first stage involved creating a sampling frame. This was developed using records from local health facilities, community health centers, and hypertension support groups within Sokode. These sources provided a database of hypertensive patients in the community. In cases where a formal registry was unavailable, healthcare professionals, including community health nurses and medical officers, were consulted to identify eligible participants from their routine interactions with patients. Eventually, the list of all possible hypertension patients were identified within the community. A total of 387 were identified as eligible for this study.

The second stage involved stratification to ensure diverse representation. The population was divided into relevant subgroups into male and female, and specific local communities (e.g., ando, Gborgame, Etoe, etc.) to capture a wide range of experiences. This stratification helped in analyzing how different demographic factors contributed to adherence levels. Based on the number of older adults and the relative location of their homes in the community, a proportional allocation of the respondents was done.

The final stage employed simple random sampling within each stratum to select participants. The list of all older adults within each stratum was created into a basket and names were selected without replacement. Participants whose names were selected were visited at their homes to assist them in responding to the questionnaire. Random sampling ensured that every eligible individual had an equal chance of being included. If some selected participants declined or were unavailable, replacements were randomly chosen from the same stratum to maintain the sample size and diversity. This multistage sampling approach provided a robust and representative sample, ensuring that the findings were generalizable to the broader population of hypertensive older adults in Sokode.

### Data collection

The data collection instrument was a self-developed questionnaire. The study and study tool consisted of three main sections. The first section gathered demographic and background information, including age, gender, marital status, educational level, occupation, and income level. It also included medical history, such as the duration of hypertension diagnosis and the type of prescribed antihypertensive medications. The second section focused on assessing participants’ knowledge of hypertensive medication. It included questions evaluating their understanding of hypertension and its treatment, the importance of taking medication as prescribed, awareness of potential consequences of non-adherence, and knowledge of lifestyle modifications such as diet and exercise. A combination of multiple-choice questions and Likert-scale items was used, with higher scores reflecting better knowledge. The third section assessed the prevalence of medication adherence. It consisted of 6 items measuring behaviors and attitudes toward medication adherence to identify factors associated with medication adherence. Questions explored individual, social, and systemic factors, including social support, perceived side effects, access to medications, healthcare provider communication, and personal health beliefs. The medication adherence scale was developed with inspiration from previous studies in the Ghanaian context (5, 25, 26). Based on these studies, the researchers basically selected only questions that were relevant in a peri-urban context to elicit medication adherence.

Trained research assistants conducted the data collection with at least a bachelor’s degree (in General Nursing or Public Health Nursing). The research assistants underwent comprehensive training on the study objectives, the questionnaire content, and ethical considerations such as obtaining informed consent and maintaining confidentiality. Data collection was conducted in private and comfortable settings to create an environment where participants felt at ease to provide honest responses. Each administration of the questionnaire took approximately 25 min. To ensure data quality, the principal investigator reviewed completed questionnaires daily, addressing any inconsistencies or omissions immediately. This process ensured that the data collected was accurate, reliable, and reflective of the participants’ experiences. All completed questionnaires were entered into Microsoft Excel 2021.

### Pretest

The questionnaire was piloted at Adaklu to assess its reliability. Feedback was gathered regarding the clarity, wording, and relevance of the questions. After analyzing the feedback, necessary revisions were made to improve the instrument. To enhance reliability, internal consistency was evaluated using Cronbach’s Alpha, with a score of 0.70 or above considered acceptable, indicating good reliability. The section relative to knowledge of hypertension and medication adherence yielded a Cronbach’s alpha score of 0.75, and the section about hypertension medication adherence had an alpha Cronbach score of 0.78.

### Data analysis

Data handling and analysis were performed using R version 4.5.1. The collected data were entered, cleaned in Microsoft Excel, and exported into R for statistical computing. Knowledge of hypertension management was assessed using nine questions, each scored on a 5-point Likert scale, yielding a maximum possible score of 45 points. Raw scores were converted to percentages and categorized as poor (0–49%), satisfactory (50–79%), or excellent (80–100%) knowledge levels. Categorical variables were presented as frequencies and percentages, while continuous knowledge scores were summarized using the mean and standard deviation. The medication adherence section yielded dichotomous responses of “Yes” and “No.” A “Yes” response was awarded a score of 1, while a no response was scored 0. Respondents who scored 4 (or more) were identified as having good medication adherence, while those who scored less than 4 were identified as having poor adherence. Bivariate analyses examining factors associated with medication adherence levels were conducted using chi-square tests, and multiple variable regression test done to identify the predictors at a statistically significant *p*-value<0.05.

### Ethical consideration

Ethical approval was obtained from the Research Ethics Committee of the University of Health and Allied Sciences (UHAS-REC A.6[48] 24-25). Informed consent was obtained from participants to take part in the study. Participants were informed of their right to either participate or withdraw from the study without any impact on their care. To ensure confidentiality, each participant was assigned a unique study number that was used in all records and data analyses. This unique identifier allowed the research team to link responses to participants without revealing their identities.

## Results

### Sociodemographic characteristics

This study enrolled 202 participants with hypertension, comprising a relatively balanced distribution of males (52.5%), aged 65–70 years (30.7%), married (39.1%) or widowed (23.8%), and nearly all had a tertiary education (43.6%). Economically, half of the participants reported low monthly income levels below 1000.00 (50.0%), and above 2000.00 (35.6%) Ghana cedis, and were unemployed (53.0%). Table 1 shows the sociodemographic characteristics of respondents.

**Table 1:** Sociodemographic characteristics.

Variables	Frequency	Percentage
Age groups (years)		
60–64	28	13.9
65–70	62	30.7
71–74	32	15.8
75–79	40	19.8
>79	40	19.8
Sex		
Female	96	47.5
Male	106	52.5
Marital status		
Married	79	39.1
Single	39	19.3
Widowed	48	23.8
Divorced	36	17.8
Educational level		
No formal education	47	23.3
Tertiary	88	43.6
Primary	24	11.9
Secondary	43	21.3
Employment status		
Unemployed	107	53.0
Employed	95	47.0
Monthly income level (gh cedis)		
<1000.00	101	50.0
1000.00–2000.00	29	14.4
>2000.00	72	35.6

### Medical history

This study’s participants exhibited substantial chronicity of hypertension, with the majority (65.8%) diagnosed for over 1 year, including living with the condition for more than a decade (37.6%). Pharmacological management was predominantly monotherapy (83.7%), with atenolol being the most prescribed antihypertensive (23.1%). The patient population demonstrated a relatively low comorbidity burden (4.5%), among which stroke was most prevalent (44.4% of those with comorbidities), followed by chronic kidney disease (22.2%). Table 2 shows the medical history.

**Table 2:** Medical history among study participants.

Variables	Frequency	Percentage
Years of diagnosis (years)		
<1	69	34.2
1–10	57	28.2
>10	76	37.6
Antihypertensive medication prescribed (*n* = 195)		
Amlodipine	19	9.7
Atenolol	45	23.1
Bendroflumethiazide	2	1
Metoprolol	22	11.3
Nifedipine	14	7.2
Lisinopril	15	7.7
Hydrochlorothiazide	9	4.6
Furosemide	25	12.8
Enalapril	31	15.9
Do not remember	13	6.7
Taking more than one antihypertensive medication		
No	169	83.7
Yes	33	16.3
Other chronic medical conditions		
No	193	95.5
Yes	9	4.5
Types of chronic medical conditions (n = 9)		
Stroke	4	44.4
Chronic Kidney disease	2	22.2
Heart failure	1	11.1
Diabetes	1	11.1
HIV	1	11.1

### Knowledge of hypertensive medication adherence among study participants

The assessment of hypertension-related knowledge among study participants revealed that the majority (80.2%) demonstrated a good level of understanding. This study revealed generally good knowledge of hypertension management among participants, with mean scores across all domains ranging from 3.37 to 3.71 on a 5-point scale. Participants demonstrated good understanding of hypertension as a lifelong condition requiring continuous treatment (mean 3.71 ± 1.27), with agreement or strongly agreeing (64.3%). Knowledge regarding correct medication dosage (mean 3.64 ± 1.21) and the risks of abruptly stopping treatment (mean 3.57 ± 1.15) was also substantial. Most participants recognized the serious complications of hypertension, including stroke and heart attack (mean 3.52 ± 1.19). Knowledge about managing medication side effects was the weakest area (mean 3.37 ± 1.16), with 20.3% reporting uncertainty about appropriate responses to adverse effects. Table 3 shows the knowledge of hypertension medication.

**Table 3:** Knowledge of hypertensive medication adherence among study participants.

Variable	Strongly disagree	Disagree	Neutral	Agree	Strongly agree	Mean ± SD
	*N*(%)	*N*(%)	*N*(%)	*N*(%)	*N*(%)	
Must take anti-hypertensive medication every day	18(8.9)	24(11.9)	45(22.3)	92(45.5)	23(11.4)	3.39 ± 1.11
High blood pressure can lead to stroke or heart attack	16(7.9)	22(10.9)	51(25.2)	66(32.7)	47(23.3)	3.52 ± 1.19
Hypertension requires lifelong treatment	14(6.9)	29(14.4)	29(14.4)	59(29.2)	71(35.1)	3.71 ± 1.27
Medication adherence helps prevent hypertension complications	13(6.4)	29(14.4)	39(19.3)	73(36.1)	48(23.8)	3.56 ± 1.18
Stopping hypertension medication suddenly can be harmful	13(6.4)	24(11.9)	44(21.8)	77(38.1)	44(21.8)	3.57 ± 1.15
Knows the correct dosage of the hypertension medication being taken	16(7.9)	19(9.4)	44(21.8)	65(32.2)	58(28.7)	3.64 ± 1.21
Knows what to do when experiencing side effects of the medication	20(9.9)	21(10.4)	56(27.7)	74(36.6)	31(15.3)	3.37 ± 1.16
Understands the consequences of skipping hypertension medication	17(8.4)	22(10.9)	59(29.2)	60(29.7)	44(21.8)	3.46 ± 1.19
Healthcare provider has explained how hypertension medications work	17(8.4)	23(11.4)	43(21.3)	72(35.6)	47(23.3)	3.54 ± 1.21

### Factors associated with good knowledge

The chi-square analysis assessing factors associated with good hypertension knowledge showed that most sociodemographic and clinical characteristics were not significantly related to knowledge levels. Age, sex, marital status, educational level, employment status, years since diagnosis, use of multiple medications, and presence of other chronic conditions all demonstrated no statistically significant associations (*p*-value > 0.05). However, monthly income showed a significant relationship with knowledge (*p*-value = 0.047), with participants earning ≤1,000 GH₵ having a higher proportion of poor knowledge compared to those earning above 1,000 GH₵ (Table 4).

**Table 4:** Chi-square analysis of factors associated with good knowledge.

Category	Bad	Good	Chi-square value	*p*-value
Age groups (years)				
60–64	5(17.9)	23(82.1)	5.258	0.262
65–70	10(16.1)	52(83.9)		
71–74	6(18.8)	26(81.2)		
75–79	6(15.0)	34(85.0)		
>79	13(32.5)	27(67.5)		
Sex				
Female	16(16.7)	80(83.3)	0.787	0.375
Male	24(22.6)	82(77.4)		
Marital status				
Married	11(13.9)	68(86.1)	2.932	0.402
Single	9(23.1)	30(76.9)		
Widowed	12(25.0)	36(75.0)		
Divorced	8(22.2)	28(77.8)		
Educational Level				
No formal education	8(17.0)	39(83.0)	0.001	0.188
Tertiary	13(14.8)	75(85.2)		
Primary	6(25.0)	18(75.0)		
Secondary	13(30.2)	30(69.8)		
Employment status				
Unemployed	17(15.9)	90(84.1)	1.702	0.192
Employed	23(24.2)	72(75.8)		
Monthly income				
≤1,000	27(26.7)	74(73.3)	6.132	**0.047**
1,001–2000	4(13.8)	25(86.2)		
>2000	9(12.5)	63(87.5)		
Years Since Diagnosis				
<1	18(26.1)	51(73.9)	4.879	0.087
1–10	6(10.5)	51(89.5)		
>10	16(21.1)	60(78.9)		
Multiple Medications				
No	30(17.8)	139(82.2)	2.006	0.157
Yes	10(30.3)	23(69.7)		
Other Chronic Conditions				
No	33(18.0)	150(82.0)	0.225	0.067
Yes	7(36.8)	12(63.2)		

Bold values indicates significant at *p* = .05.

### Predictors of good knowledge

The multivariable logistic regression analysis showed that most sociodemographic and clinical variables were not significant predictors of good knowledge of hypertension medication. Age, sex, marital status, educational level, employment status, monthly income, multiple medication use, and presence of other chronic conditions all demonstrated non-significant associations (*p*-value > 0.05). The only factor that significantly predicted good knowledge was the duration of diagnosis. Patients diagnosed for 1–10 years had three times higher odds of demonstrating good knowledge compared to those diagnosed for less than 1 year (aOR = 3.01, 95%CI:1.09–9.18, *p*-value = 0.033). Table 5 shows the predictors of good knowledge of hypertension medication.

**Table 5:** Predictors of good knowledge.

Category	aOR	95% CI	*P*-value
Age groups			
60–64 (ref)	1		
65–70	1.55	(0.42–5.30)	0.498
71–74	1.17	(0.29–4.60)	0.816
75–79	1.31	(0.32–5.19)	0.700
>79	0.6	(0.17–1.99)	0.41
Sex			
Female	1		
Male	1.04	(0.48–2.26)	0.923
Marital Status			
Married	1		
Single	0.57	(0.20–1.65)	0.299
Widowed	0.49	(0.18–1.32)	0.157
Divorced	0.57	(0.19–1.73)	0.317
Education Level			
No formal education	1		
Tertiary	1.14	(0.40–3.13)	0.800
Primary	0.81	(0.24–2.88)	0.745
Secondary	0.66	(0.22–1.92)	0.443
Employment Status			
Unemployed	1		
Employed	0.57	(0.26–1.24)	0.155
Monthly Income			
≤1,000	1		
1,001–2000	1.37	(0.44–4.99)	0.600
>2000	2.1	(0.96–5.59)	0.063
Years Since Diagnosis			
<1 year	1		
1–10 years	3.01	(1.09–9.18)	**0.033**
>10 years	1.66	(0.70–4.00)	0.252
Multiple Medications			
No	1		
Yes	0.65	(0.24–1.78)	0.388
Other Chronic Conditions			
No	1		
Yes	0.58	(0.19–1.91)	0.367

Bold values indicates significant at *p* = .05.

### Prevalence of medication adherence

Out of the total participants, 60.9% demonstrated poor adherence. The results show substantial medication non-adherence among the study participants, with approximately half reporting various forms of lapses in their antihypertensive treatment regimen. Notably, 51.0% admitted to missing at least 1 day of medication in the preceding 2 weeks, while 47.5% reported self-modifying their treatment without medical consultation due to medication-related discomfort. Concerningly, participants (50.0%) had not taken their blood pressure medication on the day prior to the survey, and some (46.5%) reported discontinuing medication when perceiving their condition as controlled. These patterns were consistent across different adherence measures, with travel-related forgetfulness (49.5%) and general treatment-related discomfort (48.5%) (Table 6).

**Table 6:** Prevalence of medication adherence.

Variables	Frequency	Percentage
In the past 2 weeks, a day occurred without taking the prescribed blood pressure medication.		
No	99	49.0
Yes	103	51.0
A reduction or cessation of the prescribed medication occurred without consulting the doctor due to feeling worse when taking it.		
No	106	52.5
Yes	96	47.5
Occasionally, medicines are not taken when traveling or away from home.		
No	102	50.5
Yes	100	49.5
The high blood pressure medication was not taken yesterday.		
No	101	50
Yes	101	50
The prescribed blood pressure medication is sometimes stopped when the condition feels under control.		
No	108	53.5
Yes	94	46.5
Following the prescribed blood pressure treatment regimen has sometimes caused discomfort.		
No	104	51.5
Yes	98	48.5

### Factors associated with medication adherence among older adult hypertensive patients

The chi-square analysis of factors associated with medication adherence among older adult hypertensive patients showed that most variables—including age, marital status, education level, employment status, years since diagnosis, use of multiple medications, and presence of other chronic conditions—were not significantly associated with adherence (*p*-value > 0.05). However, gender and monthly income were significantly associated with adherence. Male patients had better adherence than females (46.2% vs. 31.2%, *χ*^2^ = 4.137, *p*-value = 0.042), and patients with lower monthly income (<1000.00) demonstrated higher adherence (51.5%) compared to those earning 1000.00–2000.00 (31%) and >2000.00 (25%) (*χ*^2^ = 13.309, *p-*value = 0.001) (Table 7).

**Table 7:** Chi-square analysis of factors associated with medication adherence.

Variable	Category	Bad	Good	Chi-square	*p*-value
Age groups	60–64	15 (53.6%)	13(46.4%)	3.962	0.411
	65–70	43 (69.4%)	19 (30.6%)		
	71–74	21 (65.6%)	11 (34.4%)		
	75–79	22 (55%)	18 (45%)		
	>79	22 (55%)	18 (45%)		
Gender	Female	66 (68.8%)	30(31.2%)	4.137	**0.042**
	Male	57 (53.8%)	49 (46.2%)		
Marital status	Married	45 (57%)	34 (43%)	0.93	0.818
	Single	24 (61.5%)	15 (38.5%)		
	Widowed	31 (64.6%)	17 (35.4%)		
	Divorced	23 (63.9%)	13 (36.1%)		
Educational level	No formal education	33 (70.2%)	14(29.8%)	3.384	0.336
	Tertiary	54 (61.4%)	34 (38.6%)		
	Primary	12 (50%)	12 (50%)		
	Secondary	24 (55.8%)	19 (44.2%)		
Employment status	Unemployed	69 (64.5%)	38(35.5%)	0.935	0.334
	Employed	54 (56.8%)	41 (43.2%)		
Monthly income	<1000.00	49 (48.5%)	52(51.5%)	13.309	**0.001**
	1000.00–2000.00	20 (69%)	9 (31%)		
	>2000.00	54 (75%)	18 (25%)		
Years Since Diagnosis	<1	42 (60.9%)	27(39.1%)	5.094	0.078
	1–10	41 (71.9%)	16 (28.1%)		
	>10	40 (52.6%)	36 (47.4%)		
Multiple Medications	No	101 (59.8%)	68(40.2%)	0.301	0.583
	Yes	22 (66.7%)	11 (33.3%)		
Other Chronic Conditions	No	110 (60.1%)	73(39.9%)	0.211	0.646
	Yes	13 (68.4%)	6 (31.6%)		

*p* < 0.050 is statistically significant.

### Predictors of medication adherence

The analysis of factors associated with medication adherence among older adult hypertensive patients indicated that most sociodemographic characteristics—including sex, marital status, education level, employment status, and presence of other chronic diseases—were not significant predictors of adherence. The only significant associations were observed with monthly income, age (above 79 years), and years since diagnosis (more than 10 years), where patients earning between 1000.00 and 2000.00 (aOR = 0.57, 95%CI: 0.41–0.81, *p*-value = 0.002) and those earning above 2000.00 (aOR = 0.87, 95%CI: 0.41–0.90, *p*-value = 0.002) had lower odds of adherence compared to those earning less than 1000.00. Older adults aged above 79 years had higher odds of medication adherence (aOR = 2.3, 95% CI: 0.38–0.98, *p-*value = 0.050) compared to those aged 60–64 years. Those who had more than ten years of diagnosis had higher odds (oAR = 3.11, 95%CI: 1.29–1.62, *p*-value = 0.045) of medication adherence compared to those diagnosed less than a year. Table 8 shows the predictors of medication adherence.

**Table 8:** Predictors of medication adherence.

Category	Adjusted OR	95% CI	*p*-value
Age groups			
60–64	1		
65–70	1.1	0.30–6.38	0.502
71–74	1.9	0.88–1.38	0.405
75–79	3.1	0.97–4.38	0.605
>79	2.3	0.38–0.98	**0.050**
Sex			
Female	1		
Male	1.83	0.88–3.03	0.123
Marital status			
Married	1		
Single	0.9	0.68–1.17	0.423
Widowed	1.9	0.37–2.17	0.623
Divorced	2.3	0.88–7.17	0.733
Educational level			
No formal education	1		
Tertiary	1.14	0.85–1.53	0.387
Primary	4.18	0.05–6.23	0.873
Secondary	2.34	0.45–3.43	0.667
Employment status			
Unemployed	1		
Employed	0.96	0.50–1.85	0.905
Monthly income level			
<1000.00	1		
1000.00–2000.00	0.57	0.41–0.81	**0.002**
>2000.00	0.87	0.41–0.90	**0.002**
Years of diagnosis			
<1	1		
1–10	1.13	0.79–1.62	0.505
>10	3.11	1.29–1.62	**0.045**
Presence of other chronic diseases			
No	1		
Yes	0.68	0.22–2.08	0.493

Bold values indicates significant at *p* = .05.

### Reasons for non-adherence

The study identified multiple prevalent barriers to antihypertensive medication adherence, with several factors reported by nearly half or more of the participants. The most frequently endorsed barrier was being busy with other family duties (54%), being busy (generally) or late for work (47.5%), lack of reminders (51.5%), and medications being against religious or cultural beliefs (50.5%). Worry about taking medicine was also common (51.5%), as was lack of awareness that hypertension requires long-term treatment (55%). Practical challenges included drug unavailability (45.5%), financial constraints for medical expenses (49%), and forgetfulness (46%). A substantial proportion of participants reported experiencing adverse drug reactions (42.1%) and interruptions to daily routines (49%) (Table 9).

**Table 9:** Reasons for non-adherence among study participants.

Variables	Frequency	Percentage
Awareness that HPT is a long-term treatment		
No	111	55
Yes	91	45
HPT medications are against my religious and cultural beliefs		
No	100	49.5
Yes	102	50.5
Experience adverse drug reactions		
No	117	57.9
Yes	85	42.1
Belief that health depends on medicine		
No	92	45.5
Yes	110	54.5
Worry about taking medicine		
No	98	48.5
Yes	104	51.5
Forgetfulness		
No	109	54
Yes	93	46
Drug out of supply		
No	110	54.5
Yes	92	45.5
Expenses (doctors’ fees, transport, medicine, and hospitalization)		
No	103	51
Yes	99	49
Interruptions of daily routine		
No	103	51
Yes	99	49
Lack of reminders		
No	98	48.5
Yes	104	51.5
Being busy (generally) or late for work		
No	106	52.5
Yes	96	47.5
Unavailability on weekends		
No	106	52.5
Yes	96	47.5
Being busy with other family duties		
No	93	46
Yes	109	54

## Discussion

This study assessed the knowledge, prevalence, and determinants of medication adherence among older adult hypertensive patients in the Sokode community. Findings showed that knowledge of hypertension was generally high. Participants largely understood that hypertension is a lifelong condition requiring continuous treatment and were aware of the risks of treatment discontinuation. However, gaps remained regarding knowledge of the management of medication side effects and the need for consistency in daily adherence. These findings are consistent with the literature, where older adults recognize the preventive role of antihypertensive medications beyond blood pressure control to include complication mitigation (27–29). Despite the good knowledge level, medication adherence was surprisingly low, with the majority (60.9%) having poor adherence. Hypertension medication adherence ranges from 14.3 to 76.58% globally, often trending lower in resource-limited settings (12, 30). These low levels of adherence have been associated with several factors, including social, medical, health facility, cultural, and economic factors (27). Forgetfulness and treatment-related discomfort were common contributors to non-adherence (31, 32). Also, financial constraints and lack of health literacy are often emphasized as major determinants (31, 33). Unemployed individuals and those in the lower-income category were more associated with adhering to hypertension medication. These findings contrast with previous studies that showed better hypertension management was related to higher income and being employed (4, 5, 28). It was reported that higher income and active employment predict better adherence (32, 34). The association of unemployment and low economic status on medication adherence may be attributed to increased availability of time for self-management or possible reliance on subsidized medical care like that provided by the national health insurance scheme. The national health insurance (a robust social intervention policy of the government of Ghana), has assisted in providing care for people who hitherto will have had limited access due to financial barriers (35, 36). Also, relative awareness about hypertension and treatment appears to be increasing with tailored interventions targeting vulnerable populations (37). Since unemployment and longer duration of diagnosis were associated with better adherence, nurses should tailor interventions based on each patient’s lifestyle and health history, especially paying more attention to those newly diagnosed or actively working. Another reason for the association of good adherence with unemployment and low socio-economic status economic status may be due to access to subsidized care, robust family and social support systems in Ghana and the reduced requirement associated with work. Social and family support systems has been reported to have positive influence on older adults managing hypertension and on medication adherence in Ghana (5, 38, 39).

The study also identified cultural and religious beliefs as significant barriers to adherence. The association of cultural beliefs indicates a need for context-specific counseling and community engagement. Nurses should use culturally sensitive communication and involve family members of older adults when appropriate. Furthermore, interruptions to daily routines, lack of medication reminders, and competing commitments such as work were cited as barriers to non-adherence. Factors significantly associated with higher adherence included monthly income, age (above 79 years), and years since diagnosis (more than 10 years). In previous studies, however, several factors were significantly associated with medication adherence, including employment status, low income, complex treatment regimens, comorbidities, and health literacy (37, 40). In the current study, patients with hypertension for more than 10 years showed higher adherence levels, contrasting with some studies suggesting prolonged treatment duration reduces adherence due to fatigue (12). Consequently, health care providers should go beyond simply educating patients and focus more on practical support, such as teaching how to manage medication side effects and helping them set up routines or reminders (37, 41, 42). Since some patients stop taking their medicine when they feel better or change their treatment without consulting a doctor, healthcare providers, especially nurses, should provide continuous follow-up and counseling, reinforcing the need for lifelong treatment.

### Strengths and limitations

Within the Sokode community and in peri-urban communities in Ghana, this is one of the few studies focusing on older adults’ knowledge of hypertension, currently with medication adherence. Understanding knowledge levels and assessing adherence are critical for developing tailored community-based interventions that address and eliminate critical barriers. The study also used a robust system to identify by census all older adults within the Sokode community who have been diagnosed with hypertension. However, one critical limitation was the fact that this study used a self-reported questionnaire, hence increasing the risks of social desirability bias. To minimize the risk of this bias, the respondents were informed that they will be note be condemned even if their responses were not as expected and that this was research aimed at improving medication adherence. Also, older adults were required to recall their medication adherence practices (history), increasing the risk of recall bias. Consequently, ample time was given to each older adult to think about each question before responding. Furthermore, this study was a cross-sectional study that inherently has the weakness of the inability to establish a definitive causal relationship. Hence, the interpretation of the findings should be done with caution. Finally, the study was conducted in a single geographical setting, warranting that future studies may consider expanding the geographical settings to multiple places in Ghana.

## Conclusion

In this study, most older adults in Sokode showed good knowledge about hypertension medicines and understood that the disease requires regular medication. However, some did not know what to do when they experienced side effects and were not fully aware of the importance of continued daily medication. Despite this knowledge, many did not adhere to medication as prescribed. Common reasons included forgetting, stopping medication when feeling better, or changing treatment without consulting a doctor. Factors such as being unemployed, having a lower income, living with hypertension for a longer time, having more than one medication, and having excellent knowledge were linked to better adherence. Health education should not only explain the importance of taking medication but also include simple instructions on how to handle side effects, avoid stopping medication when feeling better, and understand why treatment must continue even when symptoms improve. To reduce forgetfulness and treatment interruption, healthcare workers should encourage the use of reminder tools such as alarm clocks, medication calendars, pill organizers, or support from family members. Nurses should deliver education in a way that respects local beliefs and work closely with community leaders to eliminate cultural barriers. Healthcare providers should consider simplifying medication schedules where possible and advocate for consistent availability of drugs and reduced treatment costs.

## Data Availability

The original contributions presented in the study are included in the article/supplementary material, further inquiries can be directed to the corresponding author.
